# Reduction of metastatic potential by inhibiting EGFR/Akt/p38/ERK signaling pathway and epithelial-mesenchymal transition after carbon ion exposure is potentiated by PARP-1 inhibition in non-small-cell lung cancer

**DOI:** 10.1186/s12885-019-6015-4

**Published:** 2019-08-22

**Authors:** Priyanka Chowdhury, Payel Dey, Sourav Ghosh, Asitikantha Sarma, Utpal Ghosh

**Affiliations:** 1 Department of Biochemistry & Biophysics, University of Kalyani, Kalyani, 741235 India; 2 Radiation Biology Laboratory, Inter-University Accelerator Centre, New Delhi, 110067 India

**Keywords:** Non-small-cell lung cancer, Carbon ion exposure, PARP-1, Matrix metalloproteinases (MMPs), Epithelial-mesenchymal transition (EMT), Metastatic potential

## Abstract

**Background:**

Carbon ion (^12^C) radiotherapy is becoming very promising to kill highly metastatic cancer cells keeping adjacent normal cells least affected. Our previous study shows that combined PARP-1 inhibition with ^12^C ion reduces MMP-2,-9 synergistically in HeLa cells but detailed mechanism are not clear. To understand this mechanism and the rationale of using PARP-1 inhibitor with ^12^C ion radiotherapy for better outcome in controlling metastasis, we investigated metastatic potential in two non-small cell lung cancer (NSCLC) A549 and H1299 (p53-deficient) cells exposed with ^12^C ion in presence and absence of PARP-1 inhibition using siRNA or olaparib.

**Methods:**

We monitored cell proliferation, in-vitro cell migration, wound healing, expression and activity of MMP-2, − 9 in A549 and p53-deficient H1299 cell lines exposed with ^12^C ion with and without PARP-1 inhibitor olaparib/DPQ. Expression and phosphorylation of NF-kB, EGFR, Akt, p38, ERK was also observed in A549 and H1299 cells exposed with ^12^C ion with and without PARP-1 inhibition using siRNA or olaparib. We also checked expression of few marker genes involved in epithelial-mesenchymal transition (EMT) pathways like N-cadherin, vimentin, anillin, claudin-1, − 2 in both NSCLC. To determine the generalized effect of ^12^C ion and olaparib in inhibition of cell’s metastatic potential, wound healing and activity of MMP-2, − 9 was also studied in HeLa and MCF7 cell lines after ^12^C ion exposure and in combination with PARP-1 inhibitor olaparib.

**Results:**

Our experiments show that ^12^C ion and PARP-1 inhibition separately reduces cell proliferation, cell migration, wound healing, phosphorylation of EGFR, Akt, p38, ERK resulting inactivation of NF-kB. Combined treatment abolishes NF-kB expression and hence synergistically reduces MMP-2, − 9 expressions. Each single treatment reduces N-cadherin, vimentin, anillin but increases claudin-1, − 2 leading to suppression of EMT process. However, combined treatment synergistically alters these proteins to suppress EMT pathways significantly.

**Conclusion:**

The activation pathways of transcription of MMP-2,-9 via NF-kB and key marker proteins in EMT pathways are targeted by both ^12^C ion and olaparib/siRNA. Hence, ^12^C ion radiotherapy could potentially be combined with olaparib as chemotherapeutic agent for better control of cancer metastasis.

**Electronic supplementary material:**

The online version of this article (10.1186/s12885-019-6015-4) contains supplementary material, which is available to authorized users.

## Background

Lung cancer is one of the leading causes of death of cancer patient worldwide [1]. Most of the newly diagnosed lung cancers are non-small-cell lung cancer (NSCLC) whose prognosis is very poor. Unfortunately, its survival rate is extremely low because of developing chemo-resistance and metastasis [2]. Although ionizing radiation is widely established standard radiotherapy against NSCLC, but a number of reports show the increase of malignant trait after gamma irradiation [3, 4]. However, these limitations are overcome in hadrontherapy especially using carbon ion (^12^C) which has been established as a promising modality to treat cancer [5, 6]. Epithelial-mesenchymal transition (EMT) is a key event in metastasis - the major cause of death of cancer patients. EMT is normally found during embryogenesis and is taken by cancer cells resulting proliferation of multiple malignant trait with alteration of marker genes characteristic to this process [7]. During EMT, there is secretion and activation of Matrix metalloproteinases (MMPs) that cleave extracellular matrix (ECM) to help these transformed cells to invade or migrate [8]. Unlike to gamma, ^12^C ion radiotherapy reduces invasiveness and metastatic potential although the detailed mechanisms are still unresolved [9–11].

Now-a-days, combined therapy is found to be more efficient than single mode treatment and radio-chemotherapy is becoming very promising for its better outcomes. Since Poly(ADP-ribose) polymerase-1 (PARP-1) is well-known active candidate in DNA repair, pharmacological inhibitors of PARP (iPARP) has long been used as chemosensitizer or radiosensitizer after gamma radiation [12–14]. The iPARP alone or in combination with other chemotherapeutic drugs is showing good results in clinical trials for treatment of various cancers [15–17]. Although, role of PARP-1 in metastasis is poorly understood but few reports show that PARP-1 can modulate marker genes of EMT pathway like vimentin and E-cadherin [18]. Again, iPARP alone is effective in cancer-types having defective homologous recombination (HR) pathways of repair. Can olaparib (an inhibitor of PARP-1 and PARP-2, approved after clinical trial) in combination with ^12^C ion radiotherapy be a better modality to control cancer metastasis irrespective of its efficiency in repair pathways? Actually, very few studies have been done so far combining chemotherapy with ^12^C ion radiotherapy [19, 20].

Notably, our previous report shows that combined ^12^C ion and PARP-1 inhibition induces apoptosis, reduces MMP-2/− 9 activity in a synergistic manner in HeLa cells although the detailed mechanism is not clear [20, 21]. To extend this work, we looked into cell migration, wound healing, MMP-2/− 9 activities in several human cancer cells A549, H1299, HeLa and MCF7 cells. Detailed signaling pathways of transcriptional regulation of MMP-2/− 9 and expression of key marker genes in EMT pathways were investigated in NSCLC A549 and H1299 cells after exposure with ^12^C ion in presence and absence of PARP-1 inhibition using iPARP (olaparib or DPQ) or siRNA.

## Methods

### Chemicals

Trypan blue, bovine serum albumine (BSA) and gelatin were purchased from Sigma-Aldrich, USA. Olaparib and 3, 4-dihydro-5-[4-(1-piperidinyl) butoxy]-1(2H)-isoquinolinone (DPQ) from Selleckchem, UK. Trypsin, fetal bovine serum (FBS), antibiotic solution and DMEM were purchased from HiMedia, India. The detail information about antibodies used is given in Additional file 1. Other reagents and bio-chemicals were purchased locally.

### Cell culture, treatment with iPARP and siRNA against PARP-1

Human NSCLC A549, human breast adenocarcinoma MCF7 and human cervical cancer cell HeLa, normal lung cell line L-132 were obtained from National Centre for Cell Sciences, Pune, India. H1299 (ATCC CRL-5803) cells were obtained from ATCC. All the cells were grown in DMEM medium supplemented with 10% fetal bovine serum (FBS), (complete medium) at 37 °C in a humidified incubator (5% CO_2_) [20]. The cell lines were directly used after purchase. ATCC and NCCS performs authentication of cell lines via short tandem repeat (STR) profiling analysis. So, we did not carry out additional testing to authenticate the cell lines, but its morphology and behavior were consistent with ATCC and NCCS descriptions. The cell lines were found to be without mycoplasma contamination after testing in our laboratory.

The cell lines used in this study did not require any ethical approval for their use.

The cells were treated with 1 μM of iPARP (DPQ or olaparib) 4 h before irradiation. After irradiation the cells were grown in presence of iPARP.

Equal number of cells were counted and seeded (appox. 70%) in 35 mm petridish for transfection experiment. After overnight incubation, the media were discarded and replaced with Opti-MEM, a reduced serum media (Invitrogen, life technologies). Using lipofectamine RNAiMAX Reagent (Invitrogen, life technologies) cells were transfected with Silencer Select siRNA (Ambion, life technologies) specific for PARP-1 and also with a negative control (scrambled version) according to manufacturer’s protocol. Irradiation was performed for transfected cells 48 h post transfection.

### Carbon ion (^12^C) irradiation

The cells were irradiated with ^12^C ion using the 15UD Pelletron. ^12^C ion with energy 85 MeV (equivalent to 7.08 MeV/nucleon) from the accelerator was used but the energy of ^12^C ion on the cell surface was 62 MeV (equivalent 5.16 MeV/nucleon). The corresponding entrance LET from the accelerator was 290 keV/μm, which was calculated using SRIM software. Irradiation was done following the protocol described in [19] and dose in Gray (Gy) was calculated from the particle fluence using the standard relation

$$ \mathrm{Dose}\ \left[\mathrm{Gy}\right]=1.6\times {10}^{-9}\times \mathrm{LET}\ \left[\mathrm{keV}/\upmu \mathrm{m}\right]\times \mathrm{Fluence}\ \left[\mathrm{particles}/{\mathrm{cm}}^2\right] $$

### Clonogenic cell survival

Clonogenic cell survival was done following the protocol described in [19].

### Cell proliferation assay

About 10^6^ cells were seeded in 35 mm plate and allowed to grow for 24 h. Then the cells were exposed with ^12^C ion with and without iPARP. The cells were trypsinized after 24 h of ^12^C ion exposure. Then, 3 × 10^3^ cells were seeded in 96 well plates with culture medium having 10% FBS. The plates were then incubated for 24 h, 48 h, 72 h and 96 h at 37 °C in a humidified incubator (5% CO_2_) in presence or absence of iPARP. After incubation MTT assay were performed and absorbance was measured using a microplate reader (Victor X5).

### Wound healing assay

About 1X 10^6^ number of cells were seeded on 35 mm plates and allowed to grow for 24 h in CO_2_ incubator. A scratch was made by a tip in each plate in a similar way after cells were irradiated with ^12^C ion with and without iPARP. To study the wound healing property of the cells after such treatment, the plates were photographed immediately after treatment (0 h) and after 24 h and 48 h under light microscope (Carl Zeiss, Germany). Wound healing assay was done in A549, H1299, MCF7 and in HeLa cells after treatment.

### Cell migration assay

Cell migration assay was done in A549 and H1299 cells using the protocol described in Entschladen et al. 2005 [22]. Briefly, 0.3X10^6^ cells were seeded in 35 mm petri plates and grown in complete medium for 22 h. After incubation, cells were treated with iPARP olaparib at a dose of 1 μM in serum free medium. The cells were exposed to ^12^C ion after 4 h treatment of iPARP. After irradiation, plates were incubated for 24 h in presence of iPARP. Post Incubation, medium was discarded and cells were trypsinized and counted using hemocytometer. 2 × 10^5^ cells were seeded in the upper chamber of Thin Cert™ multi-well plate having pore size of 8 μm, in serum free medium. Then, 2 ml medium containing 10% serum was added in lower chamber of the multi well plates and the cells were incubated for 19 h at at 37 °C in a humidified incubator. After incubation, medium from both the lower and upper chamber of the multi well plates was discarded and the cells in lower chamber were trypsinized and counted using hemocytometer to determine the number of cells migrated to the lower chamber from the upper porous membrane. The experiment was done in triplicates and was repeated thrice.

### Gelatin Zymography

Gelatin zymography was done in A549, H1299 and MCF7 cells according to our previously described protocol in [21]. Briefly, 0.3 × 10^6^ cells were seeded in complete medium and incubated at 37 °C for 22 h. After incubation, medium was discarded and cells were treated with 1 μM olaparib in serum free medium. Cells were irradiated with ^12^C ion 4 h post treatment of iPARP and then incubated for 24 h at 37 °C in presence of iPARP. Then, the medium was collected and centrifuged at 3000 g at 4 °C for 10 min to remove cells and other debris from the collected medium. Then the supernatant was further cleared by centrifugation with same speed. The supernatant was collected without disturbing pellet and concentrated using Amicon ultra cenrifugal protein concentrator (UFC901024, cut off 10 kDa) by centrifugation at 4600 g for 10 min at 4 °C and the total protein concentration was estimated using Victor X5 (Invitrogen). Equal amount (15 μg) of protein from each sample was resolved in 10% polyacrilamide gel containing 0.1% gelatin as a substrate. Then the gel was washed twice with 2.5x triton-X and was incubated for 19 h at 37 °C in developing buffer. The gel was then stained with Coomassie Brilliant Blue followed by overnight destaining. The gels were photographed and intensity of bands was measured using imageJ software.

### RNA extraction and Q RT- PCR

Total RNA was isolated from the treated and untreated cells using TRIzol Reagent (Invitrogen; life technologies) following standard protocol [23]. Relative expression of the genes involved in EMT pathway was studied using SYBR green master mix. The primer list is given in S1 (See Additional file 1).

### Western blot

Western blot was done following the protocol as described in [21]. Expression of MMP-2 and MMP-9 was measured both from the whole cell lysate and from the culture medium (secreted). All the secondary antibodies are HRP-conjugated and bands are developed in Luminata Crescendo western HRP substrate (Millipore) and image was taken in Chemidoc (Chemidoc XRS+ system, Biorad). The concentrations of various antibodies used are given in S2 and S3 in Additional file 1.

### Image analysis

The intensities of the gel images were determined using imageJ software [21].

### Statistical analysis

The statistical significance of the treated samples was evaluated with respect to the untreated one using ANOVA with Dunnett’s test from IBM SPSS Statistics Version 21 software. The values of significance were denoted as ‘*’ (0.01 < p ≤ 0.05), ‘**’ (0.001 < *p* ≤ 0.01) and ‘***’ (*p* ≤ 0.001). All the experiments were performed at least in triplicates.

In all figures, ‘D’ or ‘O’ means only 1 μM of DPQ or olaparib respectively; 0.5, 1, 2 represent dose of ^12^C ion in Gy; DPQ or olaparib combined with various doses of ^12^C are represented as D + 1, D + 2 etc. or O + 1, O + 2 etc.

## Results

### Clonogenic cell survival and cell proliferation

**Fig. 1:**
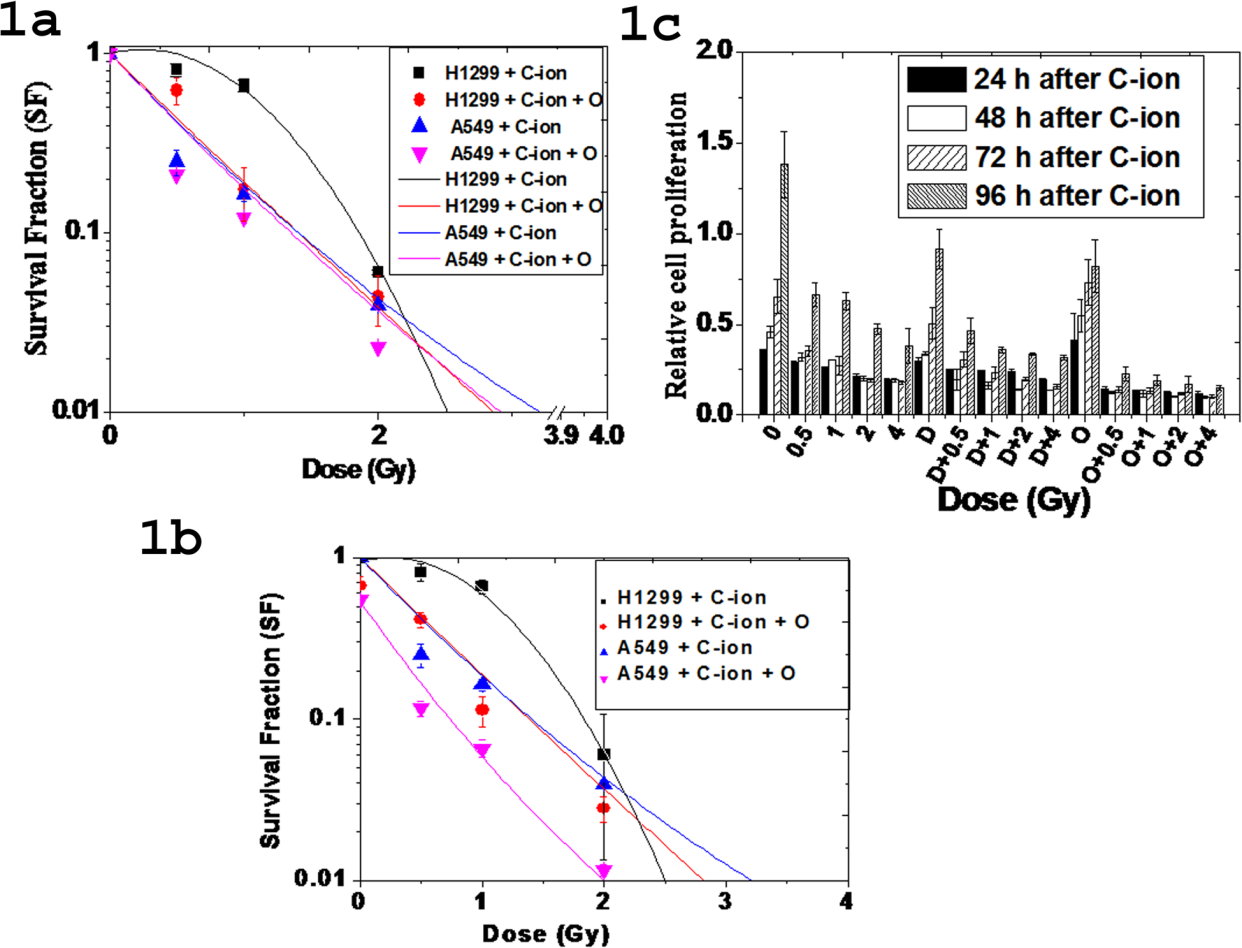
**a** Survival fraction (SF) of A549 and H1299 cells exposed with ^12^C ion with and without olaparib (O). The survival fraction (SF) of olaparib treated cells at 0 Gy was corrected to 1 and all the olaparib treated cells were normalized accordingly. **b** represent the same data where survival fraction of untreated control was assumed as 1 and of all other treated cells (either ^12^C ion or olaparib or combined) normalized accordingly. **c** Time course cell proliferation in A549 cells after treatment with PARP-1 inhibitor DPQ (D) and olaparib (O) separately and in combination with ^12^C ion exposure (0–4 Gy). Each bar represents mean cell proliferation ± standard deviation obtained from three independent experiments. All the differences with respect to control are significant and p-values at each dose were p ≤ 0.001 in each cell-type

Cell survival fraction was reduced in a dose-dependent manner in both A549 and H1299 cells treated with ^12^C ion with or without olaparib as shown in Fig. 1a. Notably, A549 cells (p53 wild-type) were more sensitive compared with H1299 (p53 mutant) cells below 2 Gy. ^12^C ion produces DNA breaks in both A549 and H1299 cells. However, wild-type p53 drives the A549 cells to cell cycle arrest and apoptosis. So, cell survival fraction (SF) is low in A549 than that of H1299 for ^12^C ion treatment only. H1299 cells escapes from cell cycle arrest and apoptosis due to absence of p53. So, the colony forming ability of H1299 is higher than that of A549 cells after ^12^C treatment only. The H1299 cells escaped from cell cycle arrest or apoptosis, fail to repair after ^12^C irradiation due to olaparib treatment and thus olaparib treatment makes H1299 cells sensitive to ^12^C ion. In Fig. 1b, we represented the data where SF of untreated control is 1 and all other treated cells (either ^12^C ion or olaparib or combined) show SF values less than 1 because each treatment gives some toxicity to the each type of cells (A549 and H1299). Only olaparib treatment reduced SF to about 35–40% in both the cell-types. But, olaparib combined with ^12^C ion reduced more than that obtained by ^12^C ion alone.

DPQ treatment alone reduced SF in a dose-dependent manner as shown in S4(B) (Additional file 1). However, DPQ combined with ^12^C ion always reduced SF more compared with single treatment. Either iPARP (olaparib or DPQ) or ^12^C ion exposure alone significantly reduced cell proliferation in a dose-dependent manner at different time interval (24 h – 96 h) as shown in Fig. 1c. Combined treatment significantly (*p* < 0.001) reduced cell proliferation at all time intervals (24 h – 96 h). Notably, olaparib sensitized A549 cells more than that obtained by DPQ within the dose range used in our experimental condition.

### Wound healing, in-vitro cell migration and activity of MMP-2,-9 by gelatin zymography assay

**Fig. 2:**
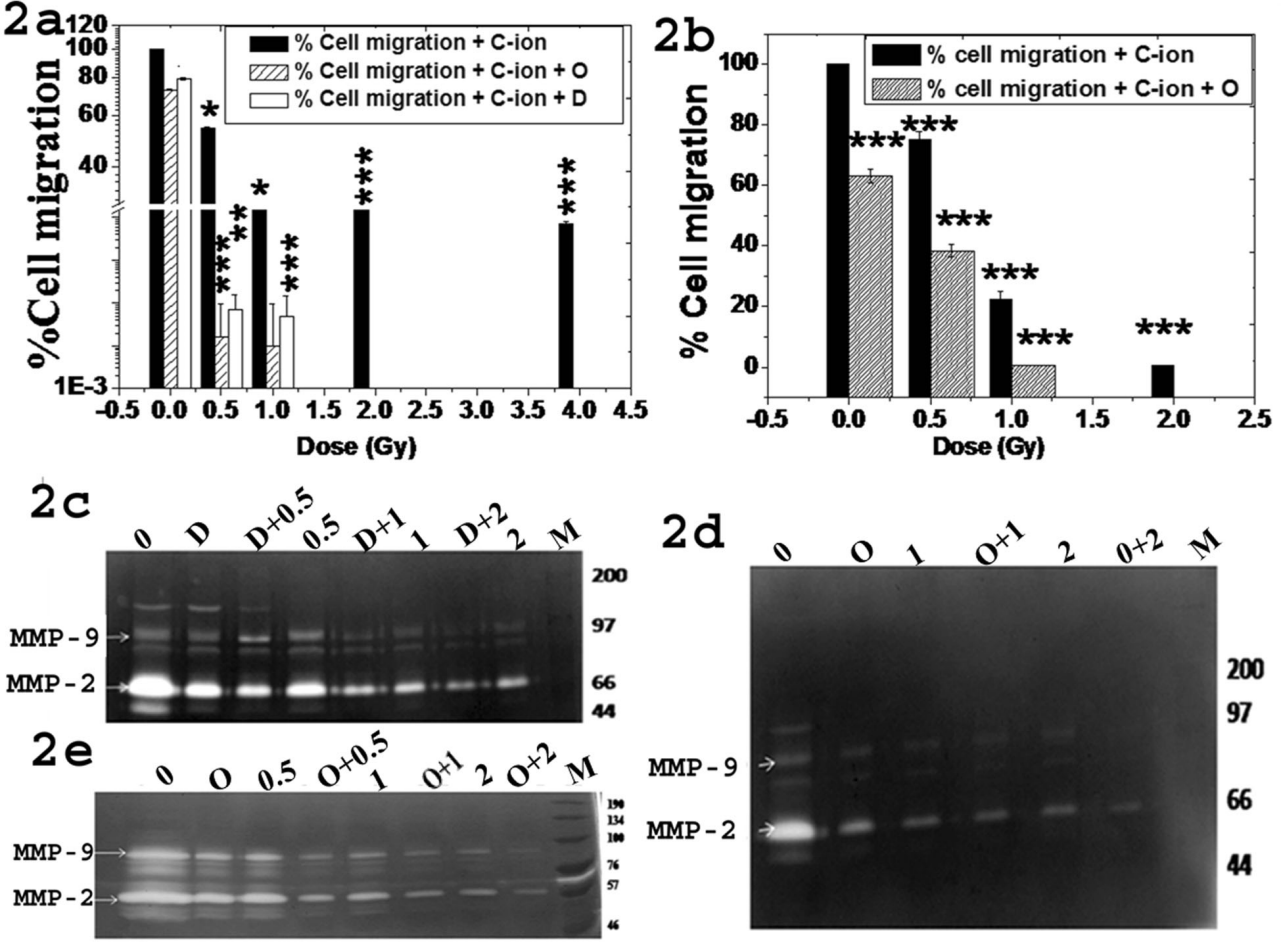
Cell migration and MMP-2, − 9 activity in A549 and H1299 cells. Percent cell migration after PARP-1 inhibition with olaparib (O), DPQ (D) and combined with ^12^C ion in A549 (**a**) and H1299 (**b**) cells. Each bar with different pattern represents mean percent cell migration with standard deviation obtained from three independent experiments in triplicates. **c**-**d**-**e** Typical photograph of gelatin zymogram to determine MMP-2 (72 kDa) and MMP-9 (92 kDa) activity after exposure with ^12^C in presence and absence of DPQ (**d**) (**c**) or olaparib (O) (**d**) in A549 cells and H1299 (**e**) cells

Both ^12^C ion and olaparib treatment alone significantly reduced wound healing in A549 (Additional file 1: S5), H1299 (Additional file 1: S6), HeLa (Additional file 1: S7) and MCF7 cells (Additional file 1: S8). Combined treatment showed synergistic effect. In-vitro cell migration was reduced dose-dependently at all doses (*p* < 0.001) in both A549 (Fig. 2a) and H1299 (Fig. 2b) cells after exposure with ^12^C ion alone. However, the in-vitro cell migration was drastically reduced to below 10% of untreated control at as low as 0.5 Gy ^12^C ion combined with 1 μM of iPARP and the cell migration was further reduced to almost nil at higher dose of ^12^C ion in presence of iPARP in both cell lines. So, combined iPARP and ^12^C ion exposure reduces wound healing and cell migration synergistically in various human cancer cells.

Activity of MMP-2 and MMP-9 was reduced to more than 45% after 1 μM of iPARP treatment alone in A549, H1299 and MCF7 cells (Additional file 1: S9). Combined treatment further reduced MMPs activity in all three cell-types as shown in Fig. 2c-e. MMP-2/− 9 activity as determined from the densitometry analysis from three independent zymogram and the loading control for each zymogram is shown in (Additional file 1: S10). This data implicates that olaparib or DPQ alone significantly reduces MMP-2, − 9 activities and combined treatment further reduces MMPs activity in all cell-types. So, reduction of MMP-2,-9 activity by single or combined treatment is not cell-specific but a generalized phenomenon irrespective of p53 status in cells.

### Signaling pathways of EGFR/Akt/p38/ERK involved in the transcriptional regulation of MMP-2 and MMP-9

**Fig. 3:**
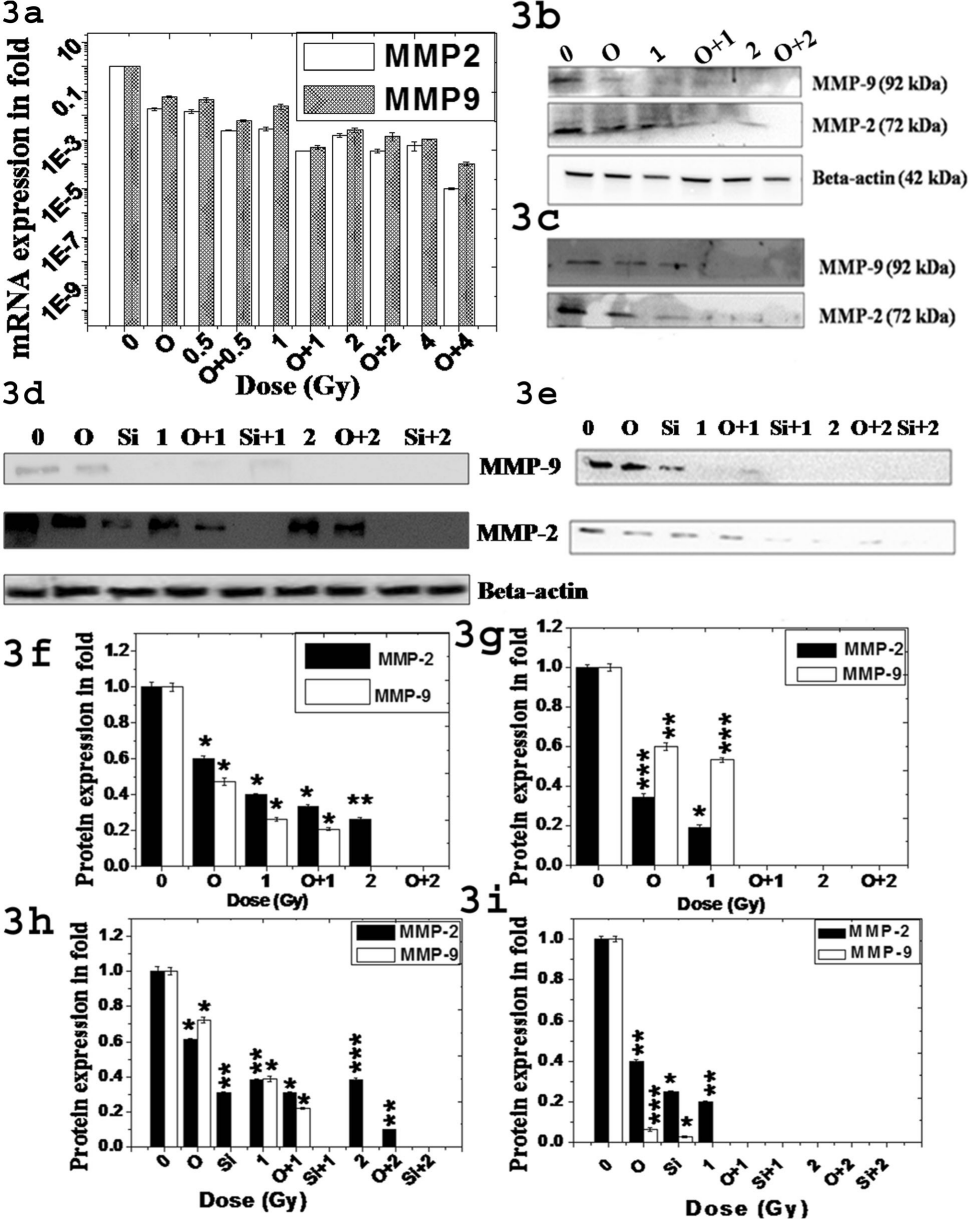
Expression of MMP-2 and MMP-9 in A549 and H1299 cells treated with ^12^C with and without olaparib (O). **a** Relative mRNA expression of MMP-2 and MMP-9 in A549 cells after 24 h treatment as determined by Real Time PCR. Each bar represents mean expression ± standard deviation obtained from three independent experiments. All the differences with respect to control are significant and p-values at each dose was p ≤ 0.001 in each cell-type. **b**-**c** & **d**-**e** Typical western blot to determine expression of MMP-2 and MMP-9 from whole cell lysate in **b** (A549) & **d** (H1299) and their respective secretion in culture medium in **c** (A549) & **e** (H1299). **f**,**g**, **h** & **i** Relative expression as measured in fold of MMP-2 and MMP-9 from whole cell lysate **f** (A549) & **h** (H1299) and their respective secretion in culture medium in **g** (A549) &  **i** (H1299)

We checked expression of these two MMPs and their secretion in culture medium during cell growth of A549 and H1299. Dose-dependent decrease of these two MMPs expression in RNA level was detected by real time PCR after single and combined treatment in A549 cells as shown in Fig. 3a. Notably, only olaparib or DPQ treatment reduced almost 40–50% expressions of these MMPs. Reduction of these MMPs were more than 80% in siPARP-1 cells (PARP-1 knockdown). We further confirmed such reduction of MMPs expression using western blot in both A549 and H1299 cells as shown in Fig. 3b and d respectively and their relative expression as measured in fold as shown in Fig. 3f and h respectively. We used total proteins secreted in serum free medium in western blot to check MMPs secretion as shown in Fig. 3c (A549) & e (H1299) respectively and their relative expression as measured in fold as shown in Fig. 3g and i respectively, which followed the same pattern as that of Fig. 3b & d. Thus, the secretion of these MMPs remained unaltered after single or combined treatment in each cell-type. However reduction of MMPs observed in medium was due to reduction of expression of those proteins inside the cells.

**Fig. 4:**
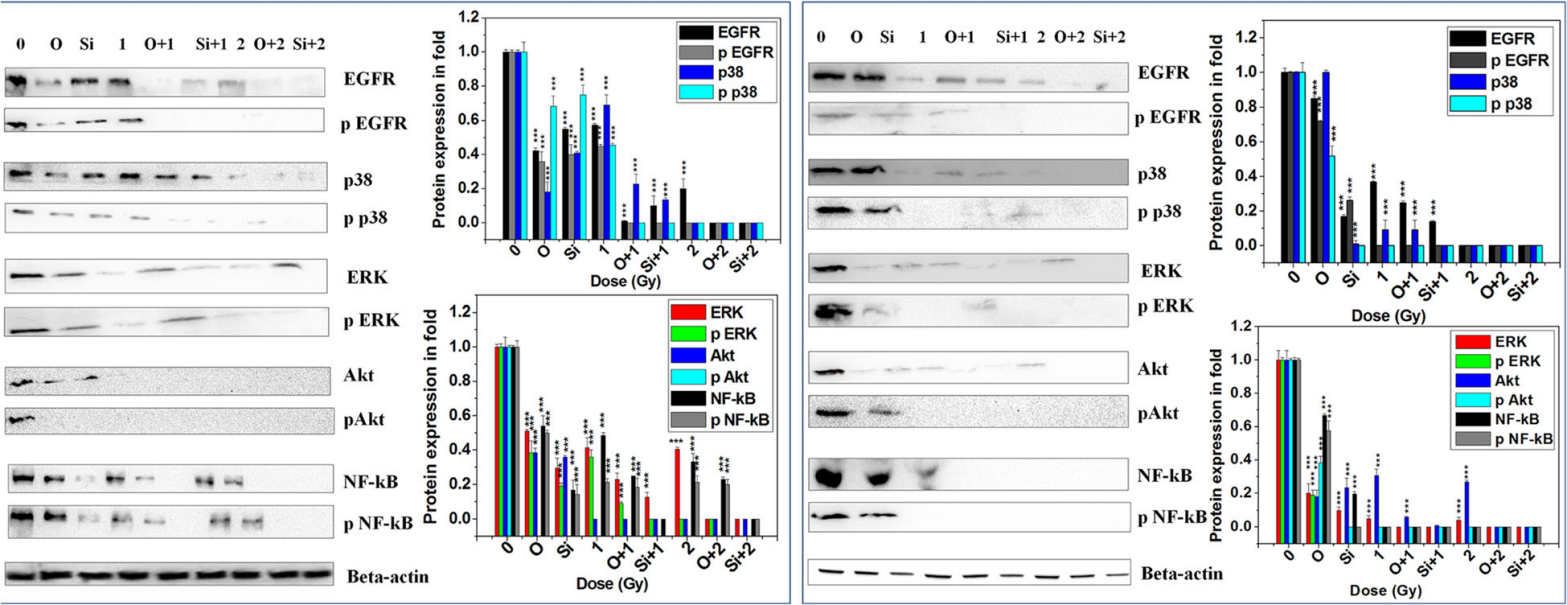
Expression and phosphorylation status of the proteins involved in the upstream signaling pathway in the transcriptional regulation of MMP-2,-9 in H1299 (left panel) and A549 (right panel) cells after ^12^C ion exposure with and without olaparib (O) or siRNA against PARP-1. The bar graphs in each panel represents the quantitative analysis of the proteins as obtained from densitometric analysis of the bands from three independent experiments using imageJ

We checked the signaling pathways of up-stream of transcriptional regulation of these MMPs in both A549 and H1299 cells as shown in Fig. 4. Treatment with iPARP or siRNA significantly reduced NF-kB expression (well-known transcription factor of MMPs) but reduction was more for siRNA. ^12^C ion exposure alone also reduced NF-kB and combined treatment just abolished NF-kB expression. Hence phospho-NF-kB was also reduced after either single treatment or combined treatment. Olaparib/siRNA or ^12^C ion treatment alone reduced all four proteins (except p38 in A549 after olaparib treatment) EGFR, ERK1/2, Akt and p38 in both A549 and H1299 cells but extent of reduction was varied in two cell-types as shown in Fig. 4. However, combined treatment always reduced synergistically all the proteins. This data implicates that PARP-1 inhibition or ^12^C ion exposure interferes with the EGFR/Akt, EGFR/p38 and EGFR/ERK1/2 signaling pathways and combined treatment completely inhibit all these pathways to inactivate NF-kB in both p53 wild-type and p53 mutant cells. Notably, olaparib treatment did not alter expression of NF-kB in normal lung cells L-132 as shown in Additional file 1. This data implicates that olaparib sensitizes cancer cells but not normal cells against ^12^C ion exposure.

### Expression of key proteins in the EMT pathways

**Fig. 5:**
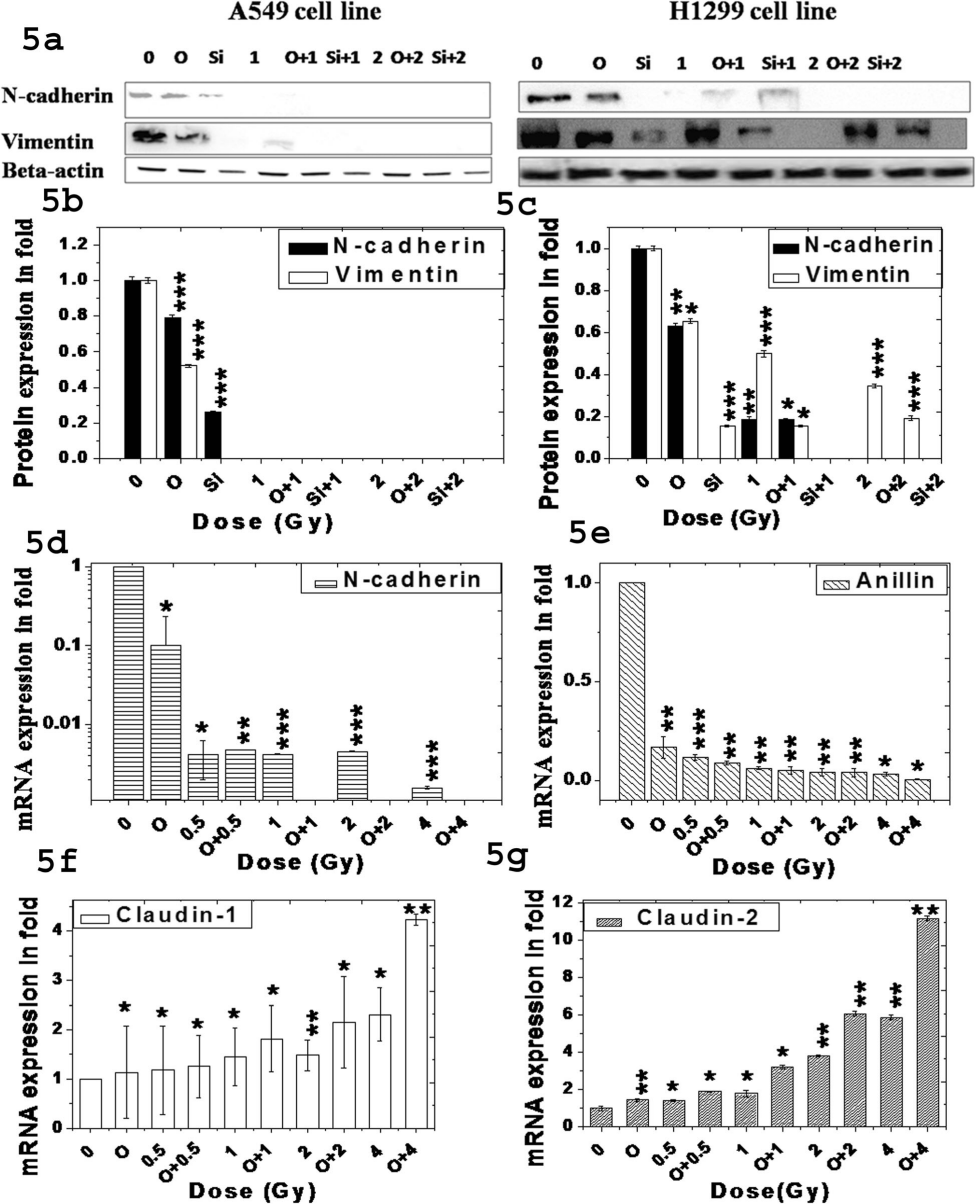
Expression of few EMT markers after ^12^C ion exposure with and without olaparib or siRNA. **a** Typical western blots of N-cadherin and vimentin in A549 (left) and H1299 (right) cells. **b** & **c** Relative expression of N-cadherin and vimentin in A549 and H1299 cells as measured in fold. **d**, **e**, **f** & **g** Expression of the EMT markers N-cadherin, anillin, claudin-1 and claudin-2 respectively by Real Time PCR after normalizing with GAPDH in A549 cell line. All the differences with respect to control are significant and p-values at each dose were p ≤ 0.001

We checked expression of key proteins involved in the EMT pathways such as N-cadherin, vimentin, anillin, claudin-1 and claudin-2 by western blot and real time PCR after single and combined treatment. N-cadherin and vimentin was decreased in A549 and H1299 cells after single or combined treatment as shown in Fig. 5a and their relative expression as measured in fold as shown in Fig. 5b and c respectively. This data was further supported by real time PCR data which indicated decrease of N-cadherin and increase of claudin-1 in RNA level after single or combined treatment as shown in Fig. 5d and f. Claudin-2 was enhanced and anillin was reduced significantly (*p* < 0.001) after treatment with olaparib or ^12^C ion separately or in combination as shown in Fig. 5g and e. This data implicates that single or combined treatment inhibits EMT pathway by altering these genes in both p53 wild-type A549 and p53 mutant H1299 cells.

## Discussion

Combined chemotherapy with radiotherapy has proven better outcomes than single mode but sufficient radiobiological information are lacking in radio-chemotherapy. Until recently, the research on iPARP has been concentrated on its role in DNA repair [24–26]. However, recent literature reports are indicating other novel functions of PARP-1 for which some of the iPARP are very much potent in vivo [27]. Incidentally, ^12^C ion reduces cell invasion and MMPs activity of various cancer cells [9, 21]. Here, we observe that PARP-1 inhibition (siRNA or iPARP) alone reduces expression and activity of MMP-2/− 9 in three cell-types and combined treatment with ^12^C ion further reduces their expression/activity. Earlier we reported that siPARP-1 HeLa cells showed reduced expression/activity of MMPs [21]. Thus reduction of MMPs expression/activity by single or combined treatment is not cell type specific. Although ^12^C ion induces cluster DNA damage and olaparib sensitizes cells to ^12^C ion. But, synergistic reduction of metastatic potential by combined treatment is not due to cytotoxicity. We observed that MMP-2, − 9 expressions were reduced about 60% and 80% after treatment with only 1 μM olaparib and 1 Gy ^12^C ion respectively after 2 h as shown in Additional file 1. However, there is no induction of apoptosis after either single treatment as shown upper panel of this file S13 in Additional file 1. Combined treatment almost abolished MMPs expression with little induction of apoptosis in terms of pro-caspase-3 activation. This data implicates that reduction of markers of metastasis is not dependent upon cytotoxicity in our experimental condition. Furthermore, Fig. 2a in our manuscript clearly shows that only olaparib treatment for 24 h reduces cell migration about 27%. But, at the same dose of olaparib the cell viability is about 90%. This data implicates that inhibition of in-vitro cell migration by single or combined treatment was not due to cytotoxicity.

Nuclear factor kappa B (NF-kB), AP1/AP2 and SP1 are known Transcription Factors (TFs) of MMP-2/− 9 [28–31]. We did not check status of AP1/AP2, SP1 here. Our data suggests that ^12^C ion exposure or PARP-1 inhibition reduces MMPs expression via reducing NF-kB in both p53 wild-type and p53 mutant cells. Functional modulation of NF-kB by PARP-1 is very complex in presence and absence of ionizing radiation and it is stimulus or cell-specific [32–34]. Other than cell proliferation and MMP’s activity, NF-kB can induce angiogenesis and anti-apoptotic genes making the cancer cells resistant to treatments [35] and hence targeting NF-kB has long been one of the good strategies for controlling cancer progression. Notably, olaparib treatment does not alter expression of NF-kB in normal lung cells L-132. In this respect, our proposed strategy of combining olaparib with ^12^C ion would target multiple pathways including NF-kB signaling to control metastatic potential of cancer cells without affecting normal cells.

Generally EGFR phosphorylation activates NF-kB via Akt, p38 or ERK1/2 pathways. Here, the expressions/phosphorylation of EGFR, Akt, p38 and ERK1/2 are reduced by iPARP / ^12^C ion separately in A549 and H1299. Hence, synergistic reduction of phosphorylation of all four proteins is observed in combined treatment to completely inactivate NF-kB. Although reduction of expression/phosphorylation of Akt and ERK1/2 by ^12^C ion is reported earlier in various cells in the context other than metastasis [36, 37], but here we report such reduction of these proteins as a part of inhibiting metastatic potential in cancer cells independent of p53 status. PARP-1 inhibition is reported to decrease phosphorylation of EGFR, p38, Akt, ERK1/2 in different cell lines [38–41]. But, our new observation is the combining olaparib with ^12^C treatment that synergistically reduces phosphorylation/activation of all four molecules leading to drastic reduction of in-vitro cell migration.

**Fig. 6:**
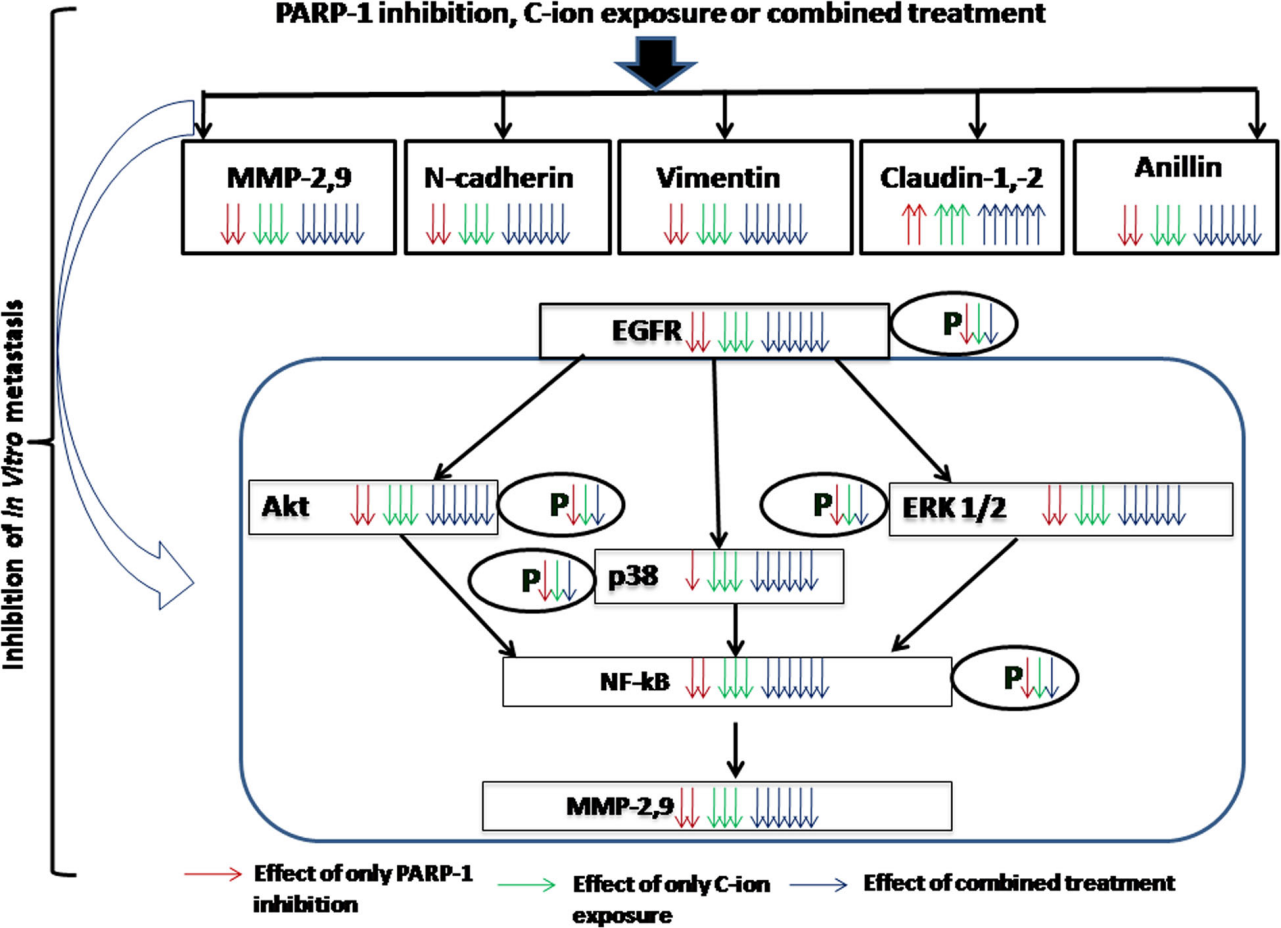
The cartoon picture represents the mechanisms of inhibition of in-vitro metastatic potential after single and combined treatment. The downward or upward arrows with three different colours beside the protein represent down-regulation or up-regulation respectively of that protein for only PARP-1 inhibition (red), only ^12^C ion exposure (green) and combined treatment (blue). Phosphorylation of each protein is denoted by P within oval shape attached with the respective protein. The down arrow of different colours beside P denotes reduction of phosphorylation of the respective proteins after different treatments

Role of ^12^C ion in EMT is largely an unexplored area. ^12^C ion reduces vimentin and anillin in different cells [9, 11]. Generally, N-cadherin, vimentin and anillin facilitates EMT whereas claudin-1, − 2 opposes EMT process. However, claudin may have complex role in metastasis [42]. Here, we report that ^12^C ion reduces N-cadherin, vimentin, anillin and increases claudin − 1, − 2 in NSCLC A549 and H1299 cells to reduce EMT process. In addition, combined ^12^C ion with PARP-1 inhibition (by siRNA or olaparib) synergistically increases claudin-1, − 2 expressions and decreases N-cadherin, vimentin, anillin. Role of PARP-1 as transcriptional modulator is well documented and it can alter expression of marker genes involved in EMT pathway [37, 43]. As such there is no report to show regulation of N-cadherin, claudin-1, − 2. Thus, decrease of N-cadherin, anillin and increase of claudin-1, − 2 by PARP-1 inhibition is our novel findings in the context of radio-chemotherapy using olaparib. So, the reduction of metastatic potential by ^12^C ion is potentiated by PARP-1 inhibition in cancer cells with wild-type or mutant p53 and the whole theme of our work is shown in Fig. 6.

## Conclusions

^12^C ion with PARP-1 inhibition by olaparib/siRNA inhibits EGFR/Akt, EGFR/p38 and EGFR/ERK1/2 signaling pathways to reduce NF-kB-mediated MMP-2/− 9 expression. Some key markers in EMT pathway are also common target of ^12^C ion and iPARP/siRNA. That’s why combined treatment synergistically reduces metastatic potential in NSCLC irrespective of p53 status in it. Our radiobiological information establishes that combining olaparib as chemotherapeutic agent with carbon ion radiotherapy would be novel approach to treat highly metastatic lung cancer.

## Additional file

Additional file 1: Reduction of in-vitro cell migration and EMT pathway in non-small lung cancer cells treated with carbon ion alone and in combination with olaparib. (DOCX 9790 kb)

## Data Availability

The datasets used and analyzed during the current study are available from the corresponding author on reasonable request.

## Abbreviations

DPQ: 3, 4-dihydro-5-[4-(1-piperidinyl) butoxy]-1(2H)-isoquinolinone
EMT: Epithelial-mesenchymal transition
Gy: Gray
MMPs: Matrix metalloproteinases
PARP: Poly(ADP-ribose) polymerase
