# Supplementary material for: Reduction of metastatic potential by inhibiting EGFR/Akt/p38/ERK signaling pathway and epithelial-mesenchymal transition after carbon ion exposure is potentiated by PARP-1 inhibition in non-small-cell lung cancer
Source: BMC Cancer. 2019 Aug 22;19:829. doi: 10.1186/s12885-019-6015-4 (PMC6704719; doi:10.1186/s12885-019-6015-4)

**S1. List of Primers used for SYBR-green q-RT PCR:**

| **Gene** | **Forward 5’-3’** | **Reverse 5’-3’** |
| --- | --- | --- |
| N- cadherin  (CDH2) | ACAGTGGCCACCTACAAAGG | CCGAGATGGGGTTGATAATGN |
| Claudin-1 (CLDN1) | ATGAGGATGGCTGTCATTGG | ATTGACTGGGGTCATAGGGT |
| Claudin-2 (CLDN2) | ATTGTGACAGCAGTTGGCTT | CTATAGATGTCACACTGGGTGAG |
| Anillin  (ANLN) | GCTGCGTAGCTTACAGACTTAC | AAGGCGTTTAAAGGTGATAGGTG |
| MMP-2 | CGCTCAGATCCGTGGTGAG | TGTCACGTGGCGTCACAGT |
| MMP-9 | CCCTGGAGACCTGAGAACCA | CCCGAGTGTAACCATAGCGG |
| GAPDH | CAATGACCCCTTCATTGACC | GATCTCGCTCCTGGAAGATG |

**S2: Details information about antibodies used:**

Primary antibodies of rabbit anti-beta actin (ab151526) and rabbit anti-MMP-2 (ab-37150) were purchased from Abcam, UK. Mouse anti-MMP-9 (sc-21733) from Santa Cruz Biotechnology, Santa Cruz, USA. Akt Antibody (9272S), Phospho-Akt (Ser473) (D9W9U) Mouse mAb (12694S), NF-κB p65 (D14E12) XP® Rabbit mAb (8242T), Phospho-NF-κB p65 (Ser536) (93H1) Rabbit mAb (3033T), p44/42 MAPK (Erk1/2) (137F5) Rabbit mAb (4695T), Phospho-p44/42 MAPK (Erk1/2) (Thr202/Tyr204) (E10)

Mouse mAb (9106S), EGF Receptor (D38B1) XP® Rabbit mAb (4267T), Phospho-EGF Receptor (Tyr1068) (1H12) Mouse mAb (2236S), p38 MAPK (D13E1) XP® Rabbit mAb (8690T), Phospho-p38 MAPK (Thr180/Tyr182) (28B10) Mouse mAb (9216S), N-Cadherin (D4R1H) XP® Rabbit mAb (13116T), Claudin-1 (D5H1D) XP® Rabbit mAb (13255T), Vimentin (D21H3) XP® Rabbit mAb (5741T) were purchased from Cell Signalling Technology (CST). HRP conjugated secondary antibody goat anti-rabbit (sc-2004) and goat anti-mouse (sc-2060) was also purchased from Santa Cruz Biotechnology, Santa Cruz, USA.

**S3. Concentrations of antibodies used in western blot:**

The concentrations of the antibodies used are as follows: MMP-2 (abcam-37150, 1:5000), MMP-9 (sc-21733, 1: 1000) and Beta-actin (ab151526, 1:10000). Expression of N-Cadherin (13116T, 1:1500), Claudin-1(13255T, 1:1500), Vimentin (5741T, 1:1500), Akt Antibody (9272S, 1:1500), Phospho-Akt (Ser473) (12694S, 1:1500), NF-κB p65 (8242T, 1:1500), Phospho-NF-κB p65 (Ser536) (3033T, 1:1500), p44/42 MAPK (Erk1/2) (4695T, 1:1500), Phospho-p44/42 MAPK (Erk1/2) (Thr202/Tyr204) (9106S, 1:1500), EGF Receptor (4267T, 1:1500), Phospho-EGF Receptor (Tyr1068) (2236S, 1:1500), p38 MAPK (8690T, 1:1500), Phospho-p38 MAPK (Thr180/Tyr182) (9216S, 1:1500) were studied. The secondary antibodies used are - goat anti-rabbit (sc-2004, 1:7000) and goat anti-mouse (sc-2060,1:5000) were HRP conjugated.

**S4. Clonogenic cell survival of A549 cells treated with ^12^C ion with and without DPQ.**

**a**

**b**

Figure S4 (a) represents the survival fraction (SF) of DPQ treated cells at 0Gy was corrected to 1 and all the DPQ treated cells were normalized accordingly. In Figure S4 (b) represented the same data where survival fraction of untreated control was 1 and all other treated cells (either ^12^C ion or DPQ or combined) showed SF values less than 1.

**S5.** Wound healing assay after 24 h and 48 h of ^12^C ion exposure in presence and absence of PARP-1 inhibitor in A549 cells. A typical picture of wound healing assay taken at 0^th^ h, 24^th^ h and 48^th^ h after ^12^C ion exposure in combination with or without olaparib (O) treatment at a dose of 1µM using 10X objective under light microscope. Here, ‘0’ means control, ‘O’ means treatment with 1µM olaparib, 0.5, 1, means 0.5 Gy and 1 Gy of ^12^C ion exposure, O+0.5, O+1 means combined treatment.


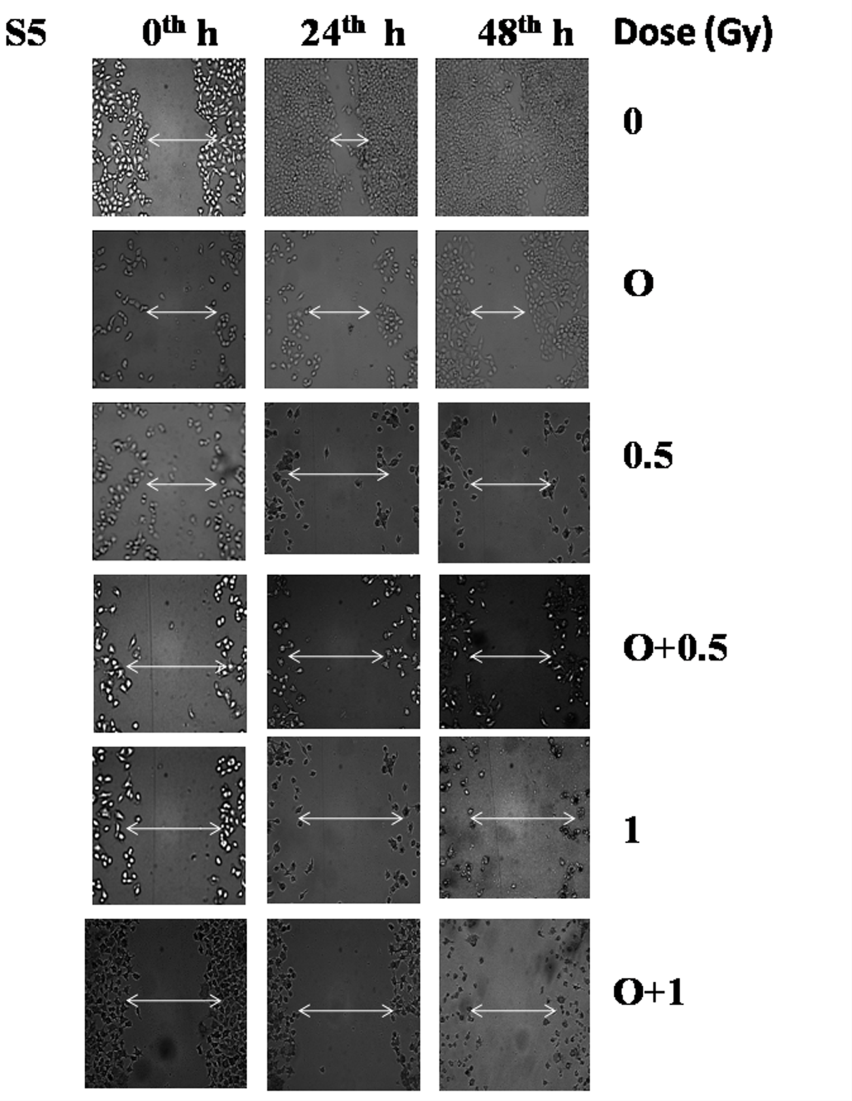


**S6.** Wound healing assay after 30 h of ^12^C ion exposure in presence and absence of PARP-1 inhibitor in H1299 cells. A typical picture of wound healing assay taken at 0^th^ h and 30^th^ h after ^12^C ion exposure in combination with or without olaparib (O) treatment at a dose of 1µM using 10X objective under light microscope. Here, ‘0’ means control, ‘O’ means treatment with 1µM olaparib, 0.5, 1, means 0.5 Gy and 1 Gy of ^12^C ion exposure, O+0.5, O+1 means combined treatment.


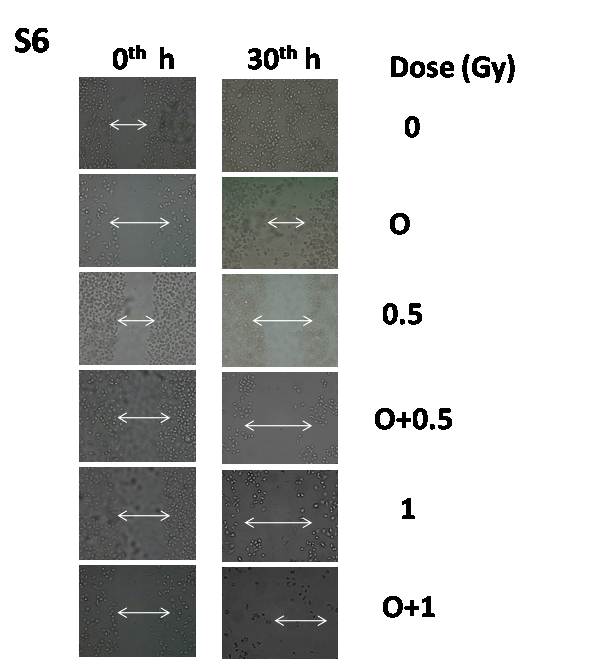


**S7.** Wound healing assay after 24 h of ^12^C ion exposure in presence and absence of PARP-1 inhibitor in HeLa cells. A typical picture of wound healing assay taken at 0^th^ h and 24^th^ h after ^12^C ion exposure in combination with or without olaparib (O) treatment at a dose of 1µM using 10X objective under light microscope. Here, ‘0’ means control, ‘O’ means treatment with 1µM olaparib, 0.5, 1, means 0.5 Gy and 1 Gy of ^12^C ion exposure, O+0.5, O+1 means combined treatment.


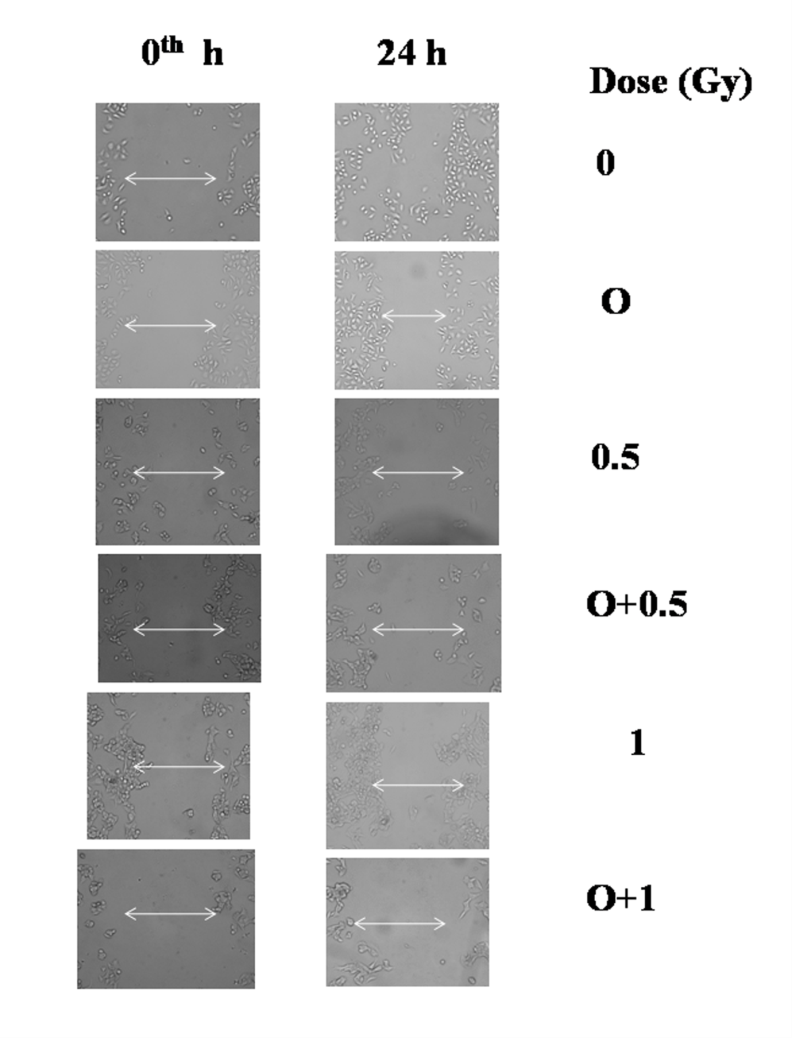


**S7**

**S8.** Wound healing assay after 48 h of ^12^C ion exposure in presence and absence of PARP-1 inhibitor in MCF7 cells. A typical picture of wound healing assay taken at 0^th^ h and 48^th^ h after ^12^C ion exposure in combination with or without olaparib (O) treatment at a dose of 1µM using 10X objective under light microscope. Here, ‘0’ means control, ‘O’ means treatment with 1µM olaparib, 0.5, 1, means 0.5 Gy and 1 Gy of ^12^C ion exposure, O+0.5, O+1 means combined treatment.


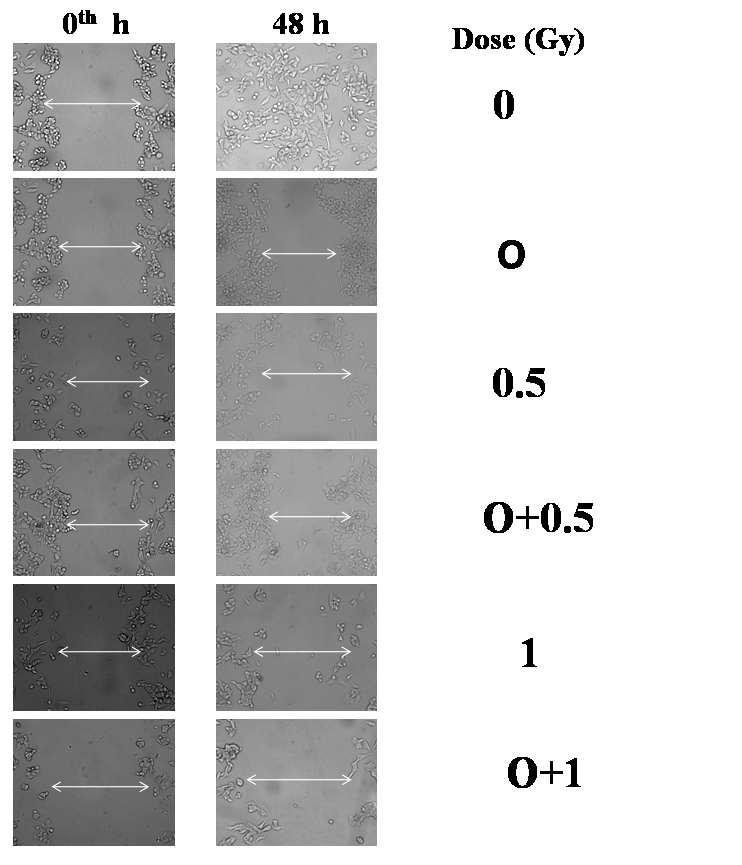


**S8**

**S9.** Typical photograph of gelatin zymogram to determine the activity of MMP-2 (72 kDa) and MMP-9 (92 kDa) after exposure with ^12^C ion in presence and absence of olaparib in MCF7 cells.

**
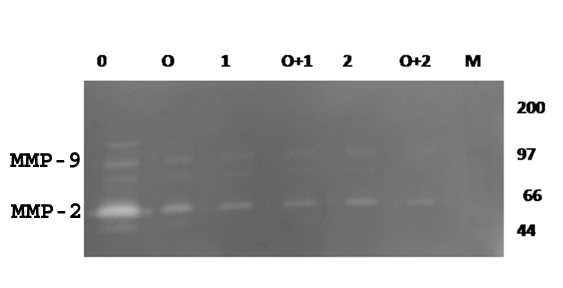
**

**S9**

**S10.** Loading control of zymogram and percent MMP-2 and MMP-9 activity as determined from the densitometry analysis using imageJ software from three independent zymogram**. a,** is the Loading control corresponding to gelatin zymogram 2c and **b**, is the loading control corresponding to gelatin zymogram 2d. C-D-E-F-G-H, Percent MMP-2 and MMP-9 activity as determined from the densitometry analysis using imageJ software from three independent zymogram. **c-d** represent MMPs activity after ^12^C ion ± DPQ (D) whereas **e-f** represent MMPs activity after ^12^C ion ± olaparib (O) in A549 cells. **g-h** represents MMPs activity after ^12^C ion ± olaparib (O) in H1299 cells.

**
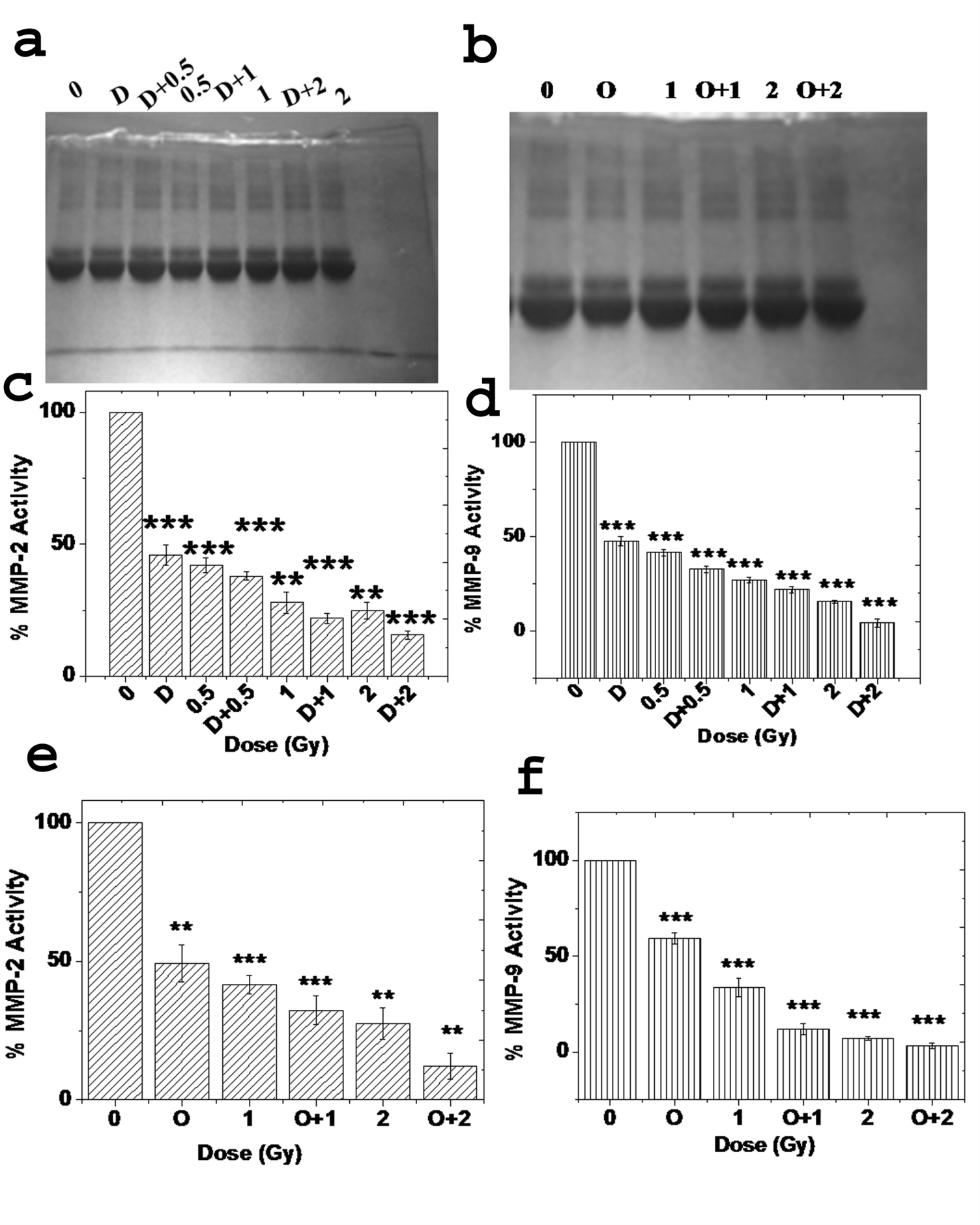
**

S10

**
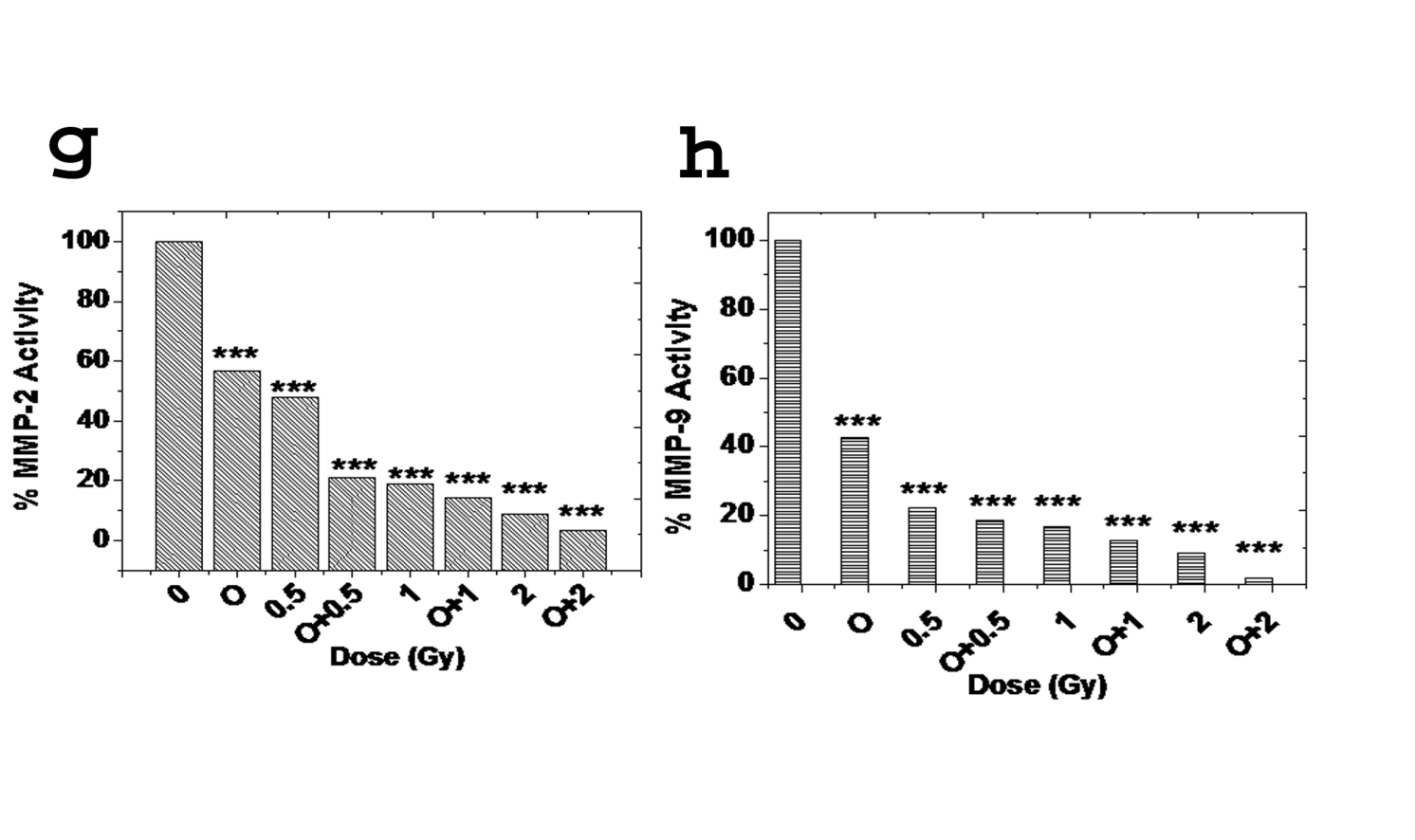
**

**S11.**

a & b - Loading control of Fig 3c using coomassie blue and Ponceau S. Here, ‘0’ means control; ‘O’ means treatment with 1µM olaparib; 1, 2 means 1 & 2 Gy of ^12^C ion exposure; O+1 & O+2 means combined 1µM olaparib with 1Gy and 2 Gy ^12^C ion exposure.

**
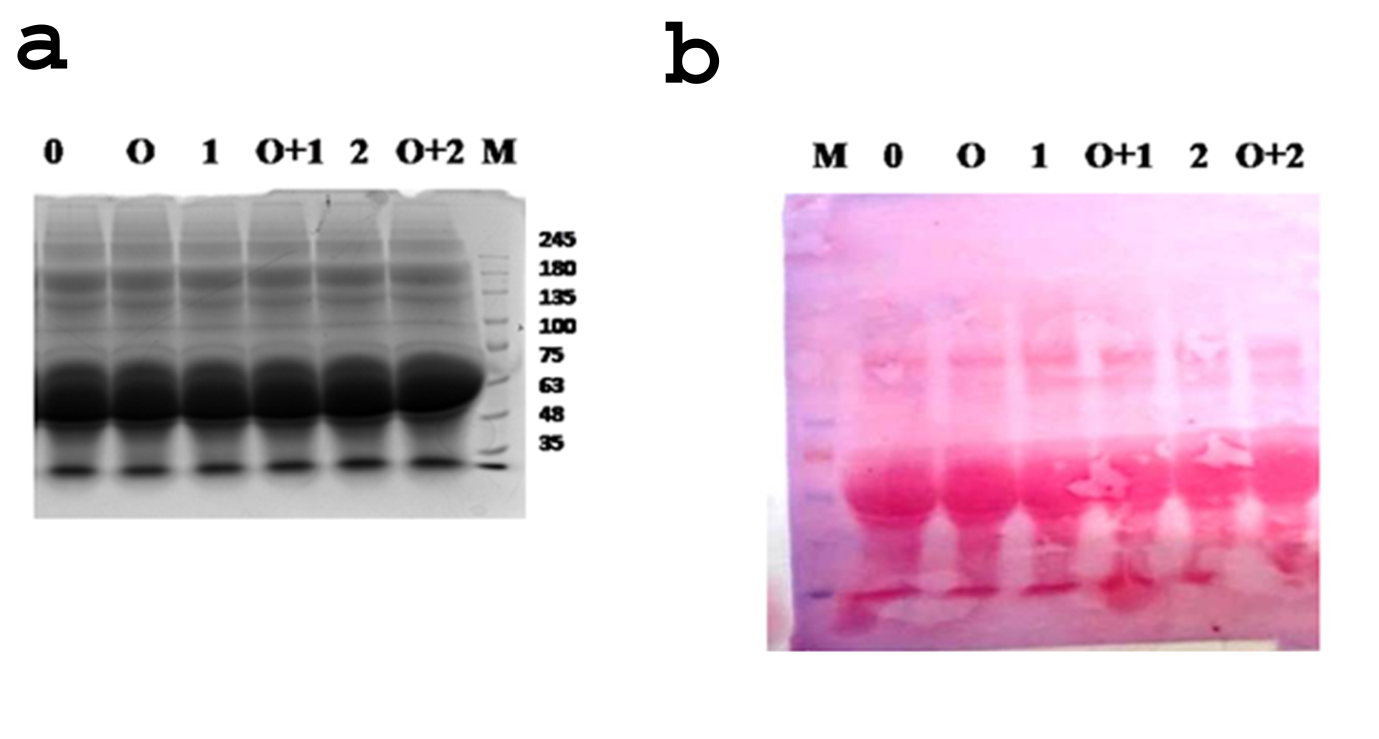
**

S11

**S12. A**, Typical western blot to determine expression of NF-kB in normal lung cell line L-132 after olaparib and BAY 11-7082 treatment. **B**, Percent cell survival of L-132 after 1µM olaparib treatment in L-132 cells for 24 hours.
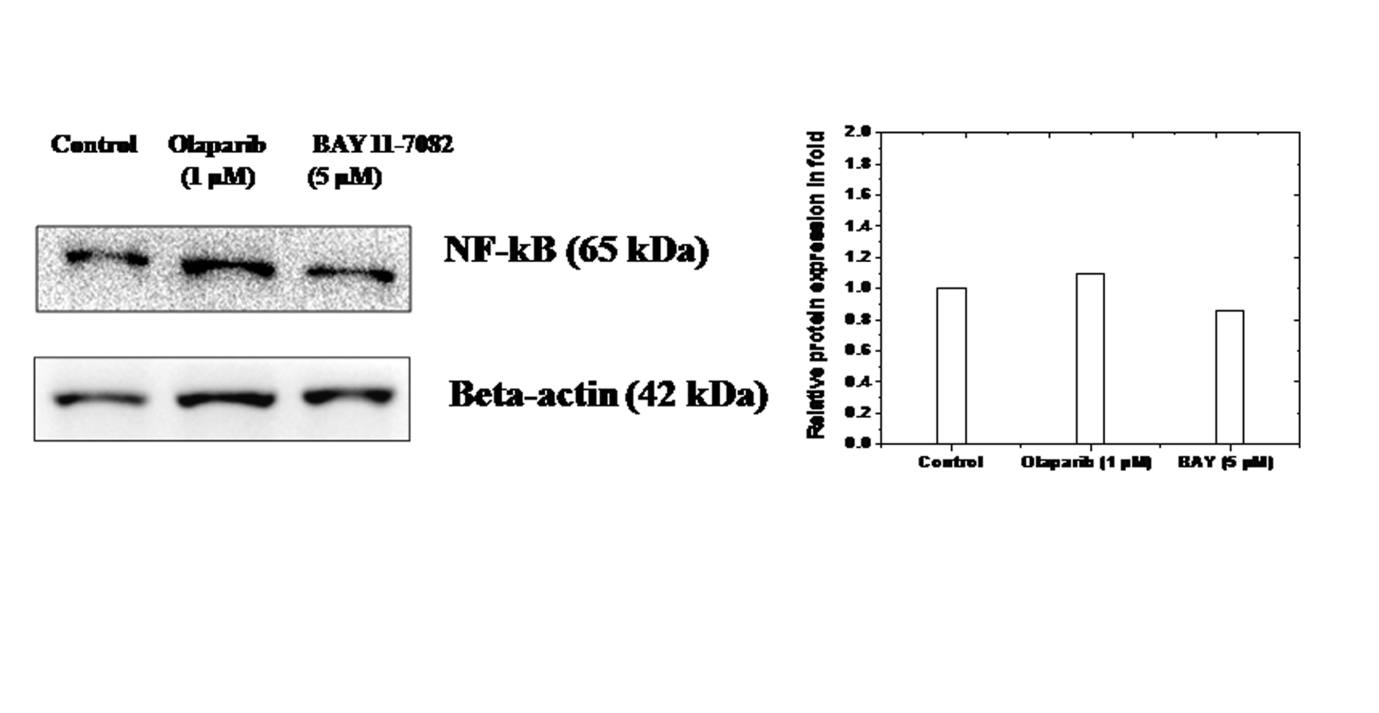


**a**

**b**

S12

**S13.** Typical western blot to determine expression of Pro-Caspase, MMP-2 and MMP-9 in A549 cells after 2 hours treatment with ^12^ C ion combined with and without olaparib (O).


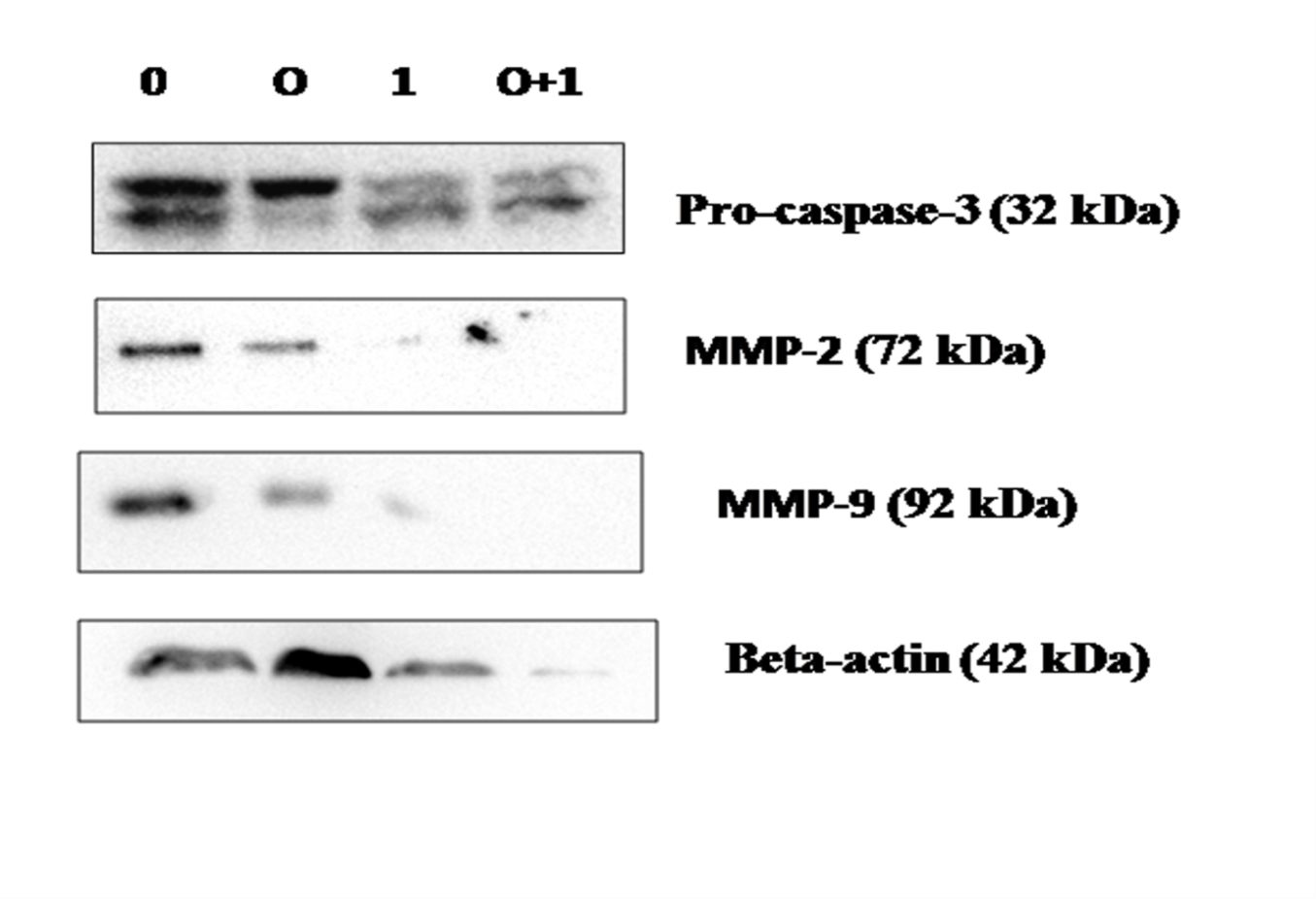


S14: Typical western blot to determine expression of claudin-1 in A549 cells after treatment with ^12^ C ion combined with and without olaparib (O).


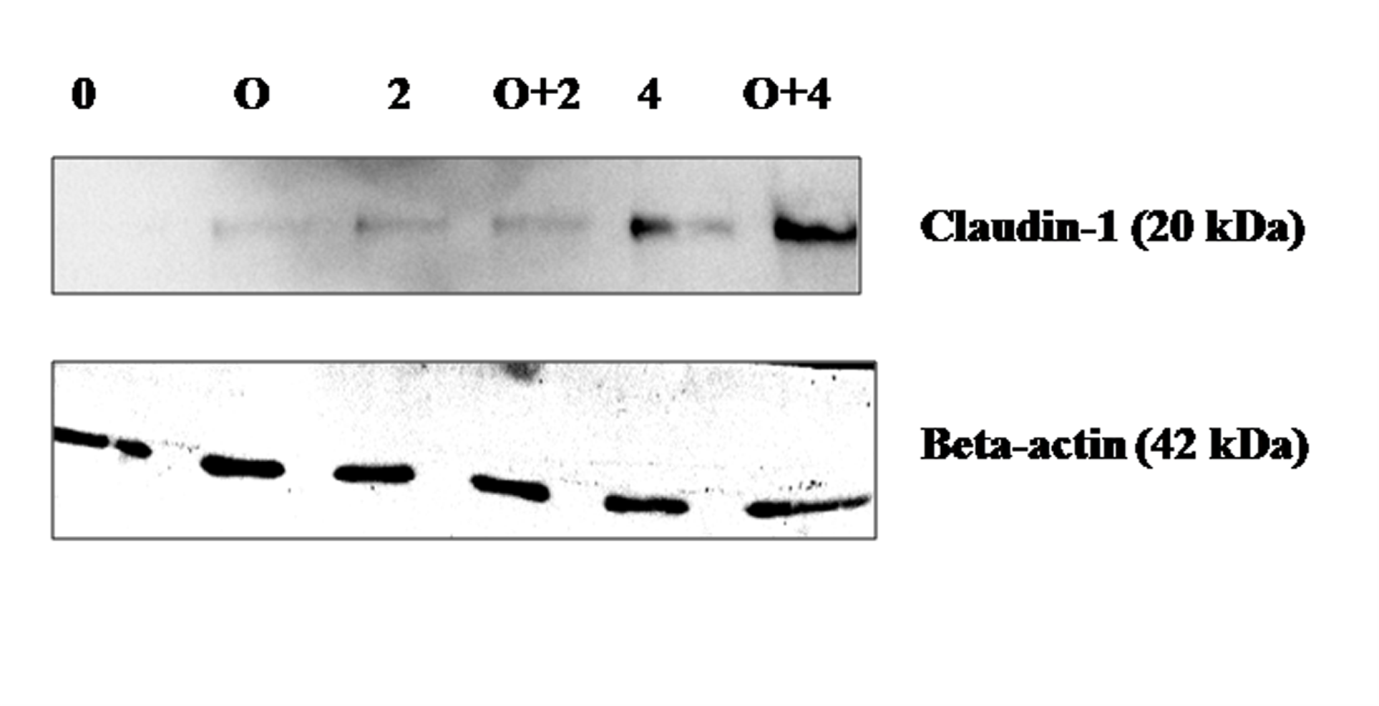

Supplement: Supplementary file 1 — Reduction of in-vitro cell migration and EMT pathway in non-small lung cancer cells treated with carbon ion alone and in combination with olaparib. (DOCX 9790 kb) [file 12885_2019_6015_MOESM1_ESM.docx]
